# Supplementary material for: The Burden of Potentially Inappropriate Medications in Chronic Polypharmacy
Source: J Clin Med. 2020 Nov 20;9(11):3728. doi: 10.3390/jcm9113728 (PMC7699788; doi:10.3390/jcm9113728)
Supplement: Supplementary file 1 [file jcm-09-03728-s001.pdf]

Journal of
Clinical Medicine

MDPI

Supplementary material : The Burden of Potentially Inappropriate Medications in Chronic Polypharmacy

Table S1. Description of Beers/Laroche criteria.

Criteria	Beers/Laroche number	ATC Used	Requirement/Exclusion
1 Antiemetics	Laroche n°30	“A03FA05” “A04AD05”	
2 First-generation antihistamines	Beers n°1 Laroche n°6;7;30	“R06AD01" "R06AE01" "R06AE51" "R06AB01" "R06AB51" "R06AA08" "R06AB04" "R06AB54" "R06AA04" "R06AA54" "R06AB06" "R06AB56" "R06AB02" "R06AB52" "R06AA09" "R06AA59" "N05BB01" "N05BB51" "R06AE05" "R06AE55" "R06AD07" "R06AD08" "R06AB05" "R06AX23" "R06AD02" "R06AD52" "R06AC01" "R03DA12" "R06AX07"	
3 Antiparkinsonian agents	Beers n°2 Laroche n°24	"N04AC01" "N04AA02" "N04AA12" "N04AA01"	
4 Anticholinergic antispasmodics	Beers n°3 Laroche n°29;8	"A03BA01" "A03CB03" "A03BA04" "A03CA02" "A03AA07" "A03AA08" "A03CB03" "A03BA03" "A03CB31" "A03BB03" "A03CB01" "G04BD04" "A03AB05" "A03CA34" "A04AD01" "N05CM05" "A04AD51" "A03BB01" "A03DB04" "G04BD08" "G04CA53" "A03AB17" "A03DA07" "G04BD07" "A03CB" “A03DB”	
5 Antipsychotic drugs	Laroche n°5	"N05AA01" "N05AB02" "N05AC01" "N05AA02" "N05AC04" "N05AA06" "N05AB03"	
6 Antithrombotics	Beers n°4;5 Laroche n°16;31	“B01AC07” “B01AC05”	Exclusion of combination Dipyridamole + aspirine extended release
7 Disopyramide	Beers n°9 Laroche n°15	C01BA03	
8 Short-acting calcium-channel blockers: nifedipine & nicardipine	Beers n°12 Laroche n°12	"C08CA05" "C08CA04"	Exclusion of extended release forms
9 Antidepressants (Tricyclic antidepressants (TCAs)/Paroxetine)	Beers n°14 Laroche n°4	N06AA09" "N06CA01" "N06AA17" "N06AA04" "N06AA01" "N06AA16" "N06AA12" "N06AA02" "N06AA21" "N06AA10" "N06AB05" "N06AA11" "N06AA06"	
10 Barbiturates	Beers n°16	"N05CA02" "N05CA01" "N03AA02" "N05CA06"	
11 Benzodiazepines Short and intermediate acting	Beers n°17 Laroche n°27	"N05BA12" "N05BA21" "N05CD04" "N05CD11" "N05BA06" "N05BA56" "N05CD06" "N05BA04" "N05CD07" "N05CD05"	

12 Benzodiazepines Long acting	Beers n°18 Laroche n°10	"N05BA08" "N05BA09" "N05BA05" "N05BA02" "N03AE01" "N05BA01" "N05CD03" "N05CD01" "N05BA18" "N05CD02" "N05BA16" "N05BA11" "N05CD10"	
13 Meprobamate	Beers n°19 Laroche n°28	"N05BC01" "N05BC51" "N05CX01"	
14 Hypnotics (z-drugs)	Beers n°20 Laroche n°27	"N05CF04" "N05CF02" "N05CF03" "N05CF01"	
15 Ergoloid mesylates	Beers n°21 Laroche n°26	"C04AE01" "C04AE51" "C04AA01" "C04AE04" "C04AE54" "N04BC03" "C04AX10" "G04BE06" "C04AX21" "C04AE02" "C04AD03" "N06BX03" "N04BC08" "C04AE54" "C05CA54" "C04AX17" "C04AX07" "C05CA51"	
16 Desiccated thyroid	Beers n°23	"H03AA05"	
17 Megestrol	Beers n°27	"L02AB01" "G03AC05" "G03DB02" "G03FA08" "G03FB04" "G03AA04" "G03AB01"	
18 Sulfonylureas - long acting	Beers n°28 Laroche n°19	"A10BB06" "A10BB02" "A10BB12" "A10BD06" "A10BD04" "A10BB01" "A10BB07"	
19 Mineral oil, given orally	Beers n°30	"A06AA01" "A06AA51" "A06AD61"	
20 Cimetidine	Laroche n°17	"A02BA01" "A02BA51"	
21 Stimulant laxatives	Laroche n°18	"A06AB02" "A06AB52" "A06AA02" "A06AG10" "A06AB05" "A06AB08" "A06AB58" "A06AB07" "A06AB57" "A06AB06" "A06AB56" "A06AB06" "A06AB56"	
22 Phenylbutazone	Laroche n°2	"M01AA01" "M02AA01" "M01BA01"	
23 Meperidine	Beers n°32	"N02AB02" "N02AG03" "N02AB52" "N02AB72"	
24 Indomethacin Ketorolac	Beers n°34 Laroche n°1	"M01AB01" "C01EB03" "M02AA23" "S01BC01" "S01CC02" "M01AB51" "M01AB15" "S01BC05" "S01FB51"	
25 Pentozocine (ex-Beers 2015)	ex Beers n°35 (Present in the 2015 version, removed in the 2019 update)	"N02AD01"	
26 Skeletal muscle relaxants	Beers n°36 Laroche n°20	"M03BA02" " M03BA52" "M03BA72" "M03BB03" "M03BB53" "M03BB73" "M03BX08" "M03BA03" "M03BA53" "M03BA73" "N04AB02" "M03BC01" "M03BC51" "M03BX07"	
27 Central alpha-agonists	Beers n°8 Laroche n°11	"C02AB" "C02LB" "C02AC01" "N02CX02" "S01EA04" "C02LC01" "C02LC51" "C02AC02" "C02AC05" "C02LC05" "C02AC06"	
28 Estrogens with or without progestins	Beers n°24	"G03CA01" "G03AA15" "G03AB07" "G03AA09" "G03AB05" "G03AA16" "G03AA12" "L02AA03" "G03AA01" "G03AA10"	Exclusion of vaginal administration

		"G03AB06" "G03AA07" "G03AB03" "G03AA03" "G03AB02" "G03AA08" "G03AA04" "G03AB01" "G03AA13" "G03AA05" "G03AB04" "G03AA11" "G03AB09" "G03AA06" "G03AA02" "G03CA03" "G03AB08" "G03CA53" "G03AA17" "G03AA14" "G03CA04" "G03CC06" "G03CA06" "G03CA07" "G03CC04" "G03CA09" "G03CA57" "G03CC07" "G03CB01" "G03CC02" "G03CB02" "G03CC05" "L02AA01" "G03CB03" "G03CC03" "G03CB04" "G03CX01" "G03FA01" "G03FB05" "G03FA02" "G03FA03" "G03FA04" "G03FA05" "G03FA06" "G03FA07" "G03FB02" "G03FA08" "G03FB04" "G03FA09" "G03FA10" "G03FB01" "G03FA11" "G03FB09" "G03FA12" "G03FB06" "G03FA13" "G03FA14" "G03FB08" "G03FA15" "G03FA16" "G03FB11" "G03FA17" "G03FB03" "G03FB07" "G03FB10" "G03FB12" "G03EA01" "G03EA02" "G03EA03" "G03HB01" "L02AA02" "L02AA04"	
29 Reserpine	Laroche n°13 Beers n°8	"C02AA02" "C02LA01" "C02LA51" "C02LA71" "C02AA52"	
30 Antipsychotics, first (conventional) and second (atypical) generation[1]	Beers n°15	"N05A"	Exclusion of Lithium (N05AN) Exclusion of patients with schizophrenia or bipolar disorder (ICD-10 : F20-29; F31; Z51.1)
31 Insulin, sliding scale[1]	Beers n°26	"A10AB"	Exclusion of fast/ intermaediate acting insulin when used at the same time than long acting insulin
32 NonsSteroidal Anti-Inflammatory Drugs (NSAIDs), oral without Pump proton inhibitor (PPI)[1]	Beers n°33	"N02BA01" "M01" "H02AA01" "H02AA02" "H02AA03" "H02AB01" "A07EA04" "H02AB02" "A01AC02" "H02AB03" "H02AB04" "H02BX01" "H02AB05" "H02AB06" "A07EA01" "A01AC54" "H02AB07" "A07EA03" "H02AB08" "A01AC01" "H02AB09" "A01AC03" "A07EA02" "H02AB10" "H02AB11" "H02AB12" "H02AB13" "H02AB14" "H02AB15" "H02AB17" "A07EA05" "A07EA06" "A07EA07" "M01BA01" "M01BA02" "M01BA03"	Exclusion of low dose aspirin. Exclusion of NSAID ued at the same time than PPI
33 Pump proton inhibitor (PPI) without chronic use of NSAID or corticosteroids[1]	Beers n°32	"A02BC"	Exclusion of PPI used at the same time than Aspirin (high dose), NSAID, corticoids
34 Psychotics in Parkinsons disease	Beers list inappropriate with	"N05A"	Parkinson patients identified by hospitalizations and long term cover with ICD 10 code : G20

	disease : Parkinson disease		Or patients with at least 3 fill/refill of a Parkinson drugs (Identified by "N04BA02" "N04BA03" "N04BC01" "N04BC02" "N04BC04" "N04BC07" "N04BD01" "N04BD02" "N04BX01" "N04BX02") Exclusion of lithium (N05AN)
35 (ex Beers 2015) Drugs innapropriate in chronic seizure	Beers list inappropriate with disease : Chronic seizure Present in 2015, removed in the 2019 update	"N06AX12" "A08AA62" "N05AA01" "N05AH02" "N06AA21" "N05AH03" "N05AC02" "N05AF04" "N02AX02" "N02AJ14" "N02AJ15" "N02AJ13"	Chronic seizure/ epilespsy patients identified by hospitalizations and long term cover with ICD 10 code : G40 - 41
36 Antipsychotics and history of fall or fractures[1]	Beers list inappropriate with disease : History of fall and fractures	"N05A"	History of fall and fractures identified by hospitalizations with ICD 10 code : W0 – 1; M84 Exclusion of lithium (N05AN)
37 Antidepressants (TCA, Selective serotonin reuptake inhibitors (SSRIs), or Serotonin–norepinephrine reuptake inhibitors (SNRIs)) with history of fall or fractures[1]	Beers list inappropriate with disease : History of fall and fractures	"N06AA" "N06AB" "G04BX14" "N06AX21" "N06AX16" "N06AX17" "N06AX23" "N06AX06"	History of fall and fractures identified by hospitalizations with ICD 10 code : W0 – 1; M
38 Opioids and history of fall or fractures[1]	Beers list inappropriate with disease : History of fall and fractures	"N02A"	History of fall and fractures identified by hospitalizations with ICD 10 code : W0 – 1; M

[1]Partially applicable criterion. PPIs: proton pump inhibitors; NSAIDs: nonsteroidal anti-inflammatory drugs, TCAs: Tricyclic antidepressants, SSRIs: Selective serotonin reuptake inhibitors, SNRIs: Serotonin–norepinephrine reuptake inhibitors.

Table S2. Description of PROMPT inappropriate criteria.

Criteria	PROMPT number	ATC Used	Requirement/Exclusion
PR0 stimulant laxatives (except if concurrent use of opioids)	PROMPT n°1	"A06AB02" "A06AB52" "A06AA02" "A06AG10" "A06AB05" "A06AB08" "A06AB58" "A06AB07" "A06AB57" "A06AB06" "A06AB56" "A06AB06" "A06AB56"	Exclusion of patients who have not received a non stimulant laxative the year before (means first line treatment; ATC: "A06AB02" "A06AB52" "A06AA02" "A06AG10" "A06AB05" "A06AB08" "A06AB58" "A06AB07" "A06AB57" "A06AB06" "A06AB56" "A06AB06" "A06AB56") Exclusion of patient who used an opioid at the same time (ATC: N02A)
PR1 Association of esomeprazole/omeprazole and clopidogrel	PROMPT n°3	"B01AC04" "A02BC01" "A02BC05"	Both inappropriate only if used at the same time (day)
PR2 Cardio-selective calcium-channel blockers and beta blockers	PROMPT n°6	"C08DB01" "C05AE03" "C08DA01" "C07A"	Both inappropriate only if used at the same time (day)
PR3 Dipyridamol – short acting	PROMPT n°7	B01AC07	Exclusion of combination Dipyridamole + aspirine extended release
PR4 First generation antihistamines	PROMPT n°8	"R06AD01" "R06AE01" "R06AE51" "R06AB01" "R06AB51" "R06AA08" "R06AB04" "R06AB54" "R06AA04" "R06AA54" "R06AB06" "R06AB56" "R06AB02" "R06AB52" "R06AA09" "R06AA59" "N05BB01" "N05BB51" "R06AE05" "R06AE55" "R06AD07" "R06AD08" "R06AB05" "R06AX23" "R06AD02" "R06AD52" "R06AC01" "R03DA12" "R06AX07"	
PR5 Theophylline in monotherapy	PROMPT n°9	"R03DA04" "R03DB04" "R03DA54" "R03DA74"	Exclusion of patients using a inhaled corticoid at the same time (ATC: "R03BA")
PR6 Oral corticoid (without use of bisphosphonate)	PROMPT n°10	"H02AA01" "H02AA02" "H02AA03" "H02AB01" "A07EA04" "H02AB02" "A01AC02" "H02AB03" "H02AB04" "H02BX01" "H02AB05" "H02AB06" "A07EA01" "A01AC54" "H02AB07" "A07EA03" "H02AB08" "A01AC01" "H02AB09" "A01AC03" "A07EA02" "H02AB10" "H02AB11" "H02AB12" "H02AB13" "H02AB14" "H02AB15" "H02AB17" "A07EA05" "A07EA06" "A07EA07" "M01BA01" "M01BA02" "M01BA03"	Exclusion of patient using a bisphosphonate at the same time (ATC: "M05BA")

PR7 Association of Selective serotonin reuptake inhibitors (SSRIs)and Venlafaxine[1]	PROMPT n°12	"N06AB" "N06AX16"	Both inappropriate only if used at the same time (day)
PR8 Tricyclic antidepressants (TCA) in first line treatment	PROMPT n°13	"N06AA"	Exclusion of patients who have received another antidepressant before (in the same year or the year before, it means first line treatment; ATC: "N06A" except N06AA)
PR91 Benzodiazepines – Short and intermediate acting	PROMPT n°14	"N05BA12" "N05BA21" "N05CD04" "N05CD11" "N05BA06" "N05BA56" "N05CD06" "N05BA04" "N05CD07" "N05CD05"	
PR92 Benzodiazepines – long acting	PROMPT n°14	"N05BA08" "N05BA09" "N05BA05" "N05BA02" "N03AE01" "N05BA01" "N05CD03" "N05CD01" "N05BA18" "N05CD02" "N05BA16" "N05BA11" "N05CD10"	
PR10 Hypnotics	PROMPT n°15	"N05CF04" "N05CF02" "N05CF03" "N05CF01"	
PR11 Carbamazepine used with Eryhtromycin/Clarithromycin	PROMPT n°16	"N03AF01"	Exclusion of day when patients received/used Eryhtromycine/Clarithromycine even if this latter was not chronic (cumulative use of at least 6 months)
PR12 Opioid (use without laxative)	PROMPT n°17	"N02A"	Exclusion of patient using a laxative at the same time (ATC: "A06")
PR13 Nitrofurantoin chronic	PROMPT n°18	"J01XE01" "J01XE51"	
PR14 Sulfonylureas - long acting	PROMPT n°19	"A10BB"	
PR15 Chronic Non-steroidal anti-inflammatory drugs (NSAIDs)	PROMPT n°20	"M01A"	
PR16 Association of two drugs among NSAIDs, Low dose aspirin or SSRI, without use of a Proton pump inhibitors (PPIs)	PROMPT n°22	"B01AC06" "N06AB" "G04BX14"	Exclusion of patient using a laxative at the same time (ATC: "A02BC"

PPIs: proton pump inhibitors; NSAIDs: nonsteroidal anti-inflammatory drugs, TCAs: Tricyclic antidepressants, SSRIs: Selective serotonin reuptake inhibitors.

Table S3. Overlaps in combined Beers/Laroche list and PROMPT list.

	Beers/ Laroche criteria from table S1	PROMPT criteria from table S2
First-generation antihistamines	2	PR4
Short-acting calcium-channel blockers: nifedipine & nicardipine	8	PR2
Tricyclic antidepressants (TCAs)	9	PR8
Benzodiazepines<br>Short and intermediate acting	11	PR91
Benzodiazepines<br>Long acting	12	PR92
Hypnotics (z-drugs)	14	PR10
Sulfonylureas - long acting	18	PR14
Stimulant laxatives	21	PR0
Non–cyclooxygenase-selective NSAIDs, oral	32 without Pump proton inhibitor (PPI)	PR15
Pump proton inhibitor (PPI) without chronic use of nonsteroidal anti-inflammatory drugs (NSAIDs) or corticosteroids	33 all PPIs without chronic use of NSAID or corticosteroids	PR1 (interaction Omeprazole clopidogrel)
Antidepressants (TCA, Selective serotonin reuptake inhibitors (SSRIs), or Serotonin–norepinephrine reuptake inhibitors (SNRIs))	37 with history of fall or fractures	PR7 Association of SSRI and Venlafaxine1
Opioids	38 with history of fall or fractures	PR12 use without laxative

PPIs: proton pump inhibitors; NSAIDs: nonsteroidal anti-inflammatory drugs, TCAs: Tricyclic antidepressants, SSRIs: Selective serotonin reuptake inhibitors, SNRIs: Serotonin–norepinephrine reuptake inhibitors.

Table S4. Prevalences of long term diseases according to chronic polypharmacy in 2016 in older adults.

Long-term diseases[1] per age class	Cohort	Chronic Polypharmacy	Chronic Hyperpolypharmacy
**Older adults (n)**	**117,545**	**27,834**	**2868**
1 - Stroke, n (%)	3639 (3.10)	1534 (5.51)	164 (5.72)
2 - Bone marrow failure and chronic cytopenia, n (%)	196 (0.17)	63 (0.23)	12 (0.42)
pl3 - Ischemic artery disease, n (%)	4727 (4.02)	2323 (8.35)	305 (10.63)
4 - Schistosomiasis, n (%)	0 (0.00)	0 (0.00)	0 (0.00)
5 – Heart failure, arrhythmia or valvular heart disease, n (%)	10,529 (8.96)	4610 (16.56)	502 (17.50)
6 – Chronic liver disease and cirrhosis, n (%)	738 (0.63)	211 (0.76)	33 (1.15)
7 - Primary immunodeficiency or HIV positive, n (%)	151 (0.13)	65 (0.23)	13 (0.45)
8 - Diabetes (type 1 or type 2), n (%)	17,020 (14.48)	8553 (30.73)	1408 (49.09)
9 – Major neuromuscular disease (including myopathy) and severe epilepsy, n (%)	959 (0.82)	333 (1.20)	47 (1.64)
10 - Haemoglobinopathy – Haemolysis – chronic hereditary or acquired and major, n (%)	24 (0.02)	6 (0.02)	1 (0.03)
11 - Haemophilia, n (%)	123 (0.10)	34 (0.12)	10 (0.35)
12 - Major high blood pressure, n (%)	6,677 (5.68)	3554 (12.77)	556 (19.39)
13 - Coronary artery disease, n (%)	9,682 (8.24)	5241 (18.83)	682 (23.78)

Long-term diseases[1] per age class	Cohort	Chronic Polypharmacy	Chronic Hyperpolypharmacy
14 – Major chronic respiratory failure, n (%)	2658 (2.26)	1183 (4.25)	267 (9.31)
15 - Alzheimer or other dementia, n (%)	4,230 (3.60)	1242 (4.46)	112 (3.91)
16 – Parkinson diseases, n (%)	1305 (1.11)	497 (1.79)	69 (2.41)
17 - Inborn errors of metabolism, n (%)	261 (0.22)	69 (0.25)	2 (0.07)
18 - Cystic fibrosis, n (%)	1 (0.00)	0 (0.00)	0 (0.00)
19 – Major chronic nephropathy and primary nephrotic syndrome, n (%)	1262 (1.07)	612 (2.20)	95 (3.31)
20 - Paraplegia, n (%)	105 (0.09)	29 (0.10)	3 (0.10)
21 - Vasculitis - Systemic lupus erythematosus - Systemic scleroderma, n (%)	539 (0.46)	205 (0.74)	22 (0.77)
22 - Rheumatoid arthritis, n (%)	1333 (1.13)	476 (1.71)	66 (2.30)
23 - Long term psychiatric disease, n (%)	3807 (3.24)	1513 (5.44)	283 (9.87)
24 – Crohn disease and ulcerative colitis, n (%)	293 (0.25)	79 (0.28)	13 (0.45)
25 - Multiple sclerosis, n (%)	165 (0.14)	54 (0.19)	6 (0.21)
26 – Scoliosis, idiopathic and evolving, n (%)	58 (0.05)	16 (0.06)	0 (0.00)
27 – Major Spondyloarthropathy, n (%)	266 (0.23)	81 (0.29)	11 (0.38)
28 – Organ transplantation follow-up, n (%)	56 (0.05)	27 (0.10)	4 (0.14)
29 – Active tuberculosis or Leprosy, n (%)	56 (0.05)	12 (0.04)	1 (0.03)
30 - Cancer or leukemia, n (%)	15,077 (12.83)	4079 (14.65)	431 (15.03)

[1]list of 30 specifically individualized chronic diseases defined by the French Health Care Insurance System.

Table 5. Prevalences of long term diseases according to polypharmacy in 2016 in middle-aged adults.

Long-term diseases[1] per age class	Cohort	Chronic Polypharmacy	Chronic Hyperpolypharmacy
**Middle-aged adults (n)**	**159,243**	**8666**	**760**
1 - Stroke, n (%)	1315 (0.83)	374 (2.71)	43 (5.66)
2 - Bone marrow failure and chronic cytopenia, n (%)	39 (0.02)	3 (0.03)	0 (0.00)
3 - Ischemic artery disease, n (%)	1599 (1.00)	478 (5.52)	57 (7.50)
4 - Schistosomiasis, n (%)	0 (0.00)	0 (0.00)	0 (0.00)
5 – Heart failure, arrhythmia or valvular heart disease, n (%)	1908 (1.20)	568 (6.55)	74 (9.74)
6 – Chronic liver disease and cirrhosis, n (%)	1109 (0.70)	159 (1.83)	16 (2.11)
7 - Primary immunodeficiency or HIV positive, n (%)	662 (0.42)	157 (1.81)	15 (1.97)
8 - Diabetes (type 1 or type 2), n (%)	9602 (6.03)	2980 (34.39)	417 (54.87)
9 – Major neuromuscular disease (including myopathy) and severe epilepsy, n (%)	1000 (0.63)	197 (2.27)	17 (2.24)
10 - Haemoglobinopathy – Haemolysis – chronic hereditary or acquired and major, n (%)	31 (0.02)	4 (0.05)	0 (0.00)
11 - Haemophilia, n (%)	110 (0.07)	19 (0.22)	1 (0.13)

Long-term diseases[1] per age class	Cohort	Chronic Polypharmacy	Chronic Hyperpolypharmacy
12 - Major high blood pressure, n (%)	1446 (0.91)	595 (6.87)	82 (10.79)
13 - Coronary artery disease, n (%)	3566 (2.24)	1545 (17.83)	167 (21.97)
14 – Major chronic respiratory failure, n (%)	1,221 (0.77)	350 (4.04)	75 (9.87)
15 - Alzheimer or other dementia, n (%)	90 (0.06)	15 (0.17)	2 (0.26)
16 – Parkinson diseases, n (%)	156 (0.10)	39 (0.45)	5 (0.66)
17 - Inborn errors of metabolism, n (%)	260 (0.16)	28 (0.32)	4 (0.53)
18 - Cystic fibrosis, n (%)	1 (0.00)	0 (0.00)	0 (0.00)
19 – Major chronic nephropathy and primary nephrotic syndrome, n (%)	477 (0.30)	169 (1.95)	22 (2.89)
20 - Paraplegia, n (%)	138 (0.09)	20 (0.23)	2 (0.26)
21 - Vasculitis - Systemic lupus erythematosus - Systemic scleroderma, n (%)	364 (0.23)	66 (0.76)	12 (1.58)
22 - Rheumatoid arthritis, n (%)	997 (0.63)	145 (1.67)	20 (2.63)
23 - Long term psychiatric disease, n (%)	6301 (3.96)	1385 (15.98)	186 (24.47)
24 – Crohn disease and ulcerative colitis, n (%)	656 (0.41)	42 (0.48)	3 (0.39)
25 - Multiple sclerosis, n (%)	438 (0.28)	60 (0.69)	9 (1.18)
26 – Scoliosis, idiopathic and evolving, n (%)	54 (0.03)	9 (0.10)	0 (0.00)
27 – Major Spondyloarthropathy, n (%)	604 (0.38)	62 (0.72)	8 (1.05)
28 – Organ transplantation follow-up, n (%)	67 (0.04)	31 (0.36)	4 (0.53)
29 – Active tuberculosis or Leprosy, n (%)	53 (0.03)	3 (0.03)	0 (0.00)
30 - Cancer or leukemia, n (%)	6914 (4.34)	688 (7.94)	61 (8.03)

[1]list of 30 specifically individualized chronic diseases defined by the French Health Care Insurance System.

Table 6. Prevalence and exposure to potentially inappropriate medications in older adults with chronic polypharmacy according to Beers criteria, Laroche list.

	Older adults with chronic polypharmacy	Older adults with chronic hyperpolypharmacy	Exposure to chronic polypharmacy in older adults (%)
	**n = 27,834**	**n = 2868**	**100**
Potentially inappropriate medication - *broad*[1]	18,036 (64.8)	2544 (88.7)	13.5
Potentially inappropriate medication-*narrow*[2]	10,220 (36.7)	1730 (60.3)	6.7
Beers/Laroche criteria			
1 Antiemetics	0 (0.0)	0 (0.0)	0.0
2 First-generation antihistamines	659 (2.4)	159 (5.5)	0.3
3 Antiparkinsonian agents	133 (0.5)	21 (0.7)	0.1
4 Anticholinergic antispasmodics	626 (2.3)	119 (4.2)	0.3
5 Antipsychotic drugs	223 (0.8)	47 (1.6)	0.1
6 Antithrombotics	23 (0.1)	4 (0.1)	< 0.1

	Older adults with chronic polypharmacy	Older adults with chronic hyperpolypharmacy	Exposure to chronic polypharmacy in older adults (%)
	n = 27,834	n = 2868	100
7 Disopyramide	23 (0.1)	0 (0.0)	< 0.1
8 Short-acting calcium-channel blockers: nifedipine & nicardipine	19 (0.1)	5 (0.2)	0.0
9 Antidepressants (Tricyclic antidepressants (TCAs)/Paroxetine)	1324 (4.8)	228 (8.0)	0.7
10 Barbiturates	87 (0.3)	10 (0.4)	0.1
11 Benzodiazepines Short and intermediate acting	3807 (13.7)	660 (23.0)	2.0
12 Benzodiazepines Long acting	1271 (4.6)	286 (10.0)	0.6
13 Meprobamate	0 (0.0)	0 (0.0)	0.0
14 Hypnotics (z-drugs)	1688 (6.1)	382 (13.3)	0.8
15 Ergoloid mesylates	380 (1.4)	71 (2.5)	0.2
16 Desiccated thyroid	0 (0.0)	0 (0.0)	0.0
17 Megestrol	0 (0.0)	0 (0.0)	0.0
18 Sulfonylureas - long acting	1071 (3.9)	201 (7.0)	0.6
19 Mineral oil, given orally	205 (0.7)	39 (1.4)	0.1
20 Cimetidine	3 (0.0)	0 (0.0)	0.0
21 Stimulant laxatives	1 (0.0)	0 (0.0)	0.0
22 Phenylbutazone	0 (0.0)	0 (0.0)	0.0
23 Meperidine	0 (0.0)	0 (0.0)	0.0
24 Indomethacin Ketorolac	24 (0.1)	6 (0.2)	< 0.1
25 Pentozocine (ex-Beers 2015)	0 (0.0)	0 (0.0)	0.0
26 Skeletal muscle relaxants	0 (0.0)	0 (0.0)	0.0
27 Central alpha-agonists	1404 (5.0)	308 (10.7)	0.8
28 Estrogens with or without progestins	181 (0.7)	35 (1.2)	0.1
29 Reserpine	0 (0.0)	0 (0.0)	0.0
30 Antipsychotics, first (conventional) and second (atypical) generation[3]	227 (0.8)	43 (1.5)	0.1
31 Insulin, sliding scale[1]	108 (0.4)	40 (1.4)	< 0.1
32 NonsSteroidal Anti-Inflammatory Drugs (NSAIDs), oral without Pump proton inhibitor (PPI)[3]	418 (1.5)	65 (2.3)	0.2
33 Pump proton inhibitor (PPI) without chronic use of NSAID or corticosteroids[3]	12,073 (43.4)	1924 (67.1)	6.3
34 Psychotics in Parkinsons disease	66 (0.2)	10 (0.4)	< 0.1
35 (ex Beers 2015) Drugs innapropriate in chronic seizure	34 (0.1)	7 (0.2)	< 0.1
36 Antipsychotics and history of fall or fractures[3]	86 (0.3)	14 (0.5)	< 0.1
37 Antidepressants (TCA, Selective serotonin reuptake inhibitors (SSRIs), or Serotonin–norepinephrine reuptake inhibitors (SNRIs)) with history of fall or fractures[3]	420 (1.5)	73 (2.6)	0.2
38 Opioids and history of fall or fractures[3]	134 (0.5)	35 (1.2)	0.1

[1]considering both fully and partially applicable criteria [2]considering only fully applicable criteria [3] partially applicable criteria, [1,2,3]more details are provided in table S1. PPIs: proton pump inhibitors; NSAIDs: nonsteroidal anti-inflammatory drugs, TCAs: Tricyclic antidepressants, SSRIs: Selective serotonin reuptake inhibitors, SNRIs: Serotonin–norepinephrine reuptake inhibitors.

Table 7. Prevalence and exposure to potentially inappropriate medications and interactions in middle-aged adults with chronic polypharmacy according to PROMPT criteria.

	Middle-aged adults with chronic polypharmacy	Middle-aged adults with chronic hyperpolypharmacy	Exposure to chronic polypharmacy in middle-aged adults (%)
	n = 8,666	n = 760	100
Potentially inappropriate medication	4009 (46.2)	570 (75.0)	10.4
PR0 stimulant laxatives (except if concurrent use of opioids)	0 (0.0)	0 (0.0)	0.0
PR1 Association of esomeprazole/omeprazole and clopidogrel	251 (2.9)	59 (7.8)	0.8
PR2 Cardio-selective calcium-channel blockers and beta blockers	28 (0.3)	4 (0.5)	0.1
PR3 Dipyridamol – short acting	2 (0.0)	0 (0.0)	0.0
PR4 First generation antihistamines	450 (5.2)	90 (11.8)	0.7
PR5 Theophylline in monotherapy	15 (0.2)	4 (0.5)	< 0.1
PR6 Oral corticoid (without use of bisphosphonate)	176 (2.0)	38 (5.0)	0.3
PR7 Association of Selective serotonin reuptake inhibitors (SSRIs)and Venlafaxine[1]	15 (0.2)	1 (0.1)	< 0.1
PR8 Tricyclic antidepressants (TCA) in first line treatment	107 (1.2)	16 (2.1)	0.2
PR91 Benzodiazepines – Short and intermediate acting	1395 (16.1)	232 (30.5)	2.7
PR92 Benzodiazepines – long acting	879 (10.1)	138 (18.2)	1.5
PR10 Hypnotics	637 (7.4)	115 (15.1)	1.0
PR11 Carbamazepine used with Eryhtromycin/Clarithromycin	2 (0.0)	1 (0.0)	0.0
PR12 Opioid (use without laxative)	639 (7.4)	143 (18.8)	1.1
PR13 Nitrofurantoin chronic	0 (0.0)	0 (0.0)	0.0
PR14 Sulfonylureas - long acting	1069 (12.3)	178 (23.4)	1.9
PR15 Chronic Non-steroidal anti-inflammatory drugs (NSAIDs)	80 (0.9)	14 (1.8)	0.1
PR16 Association of two drugs among NSAIDs, Low dose aspirin or SSRI, without use of a Proton pump inhibitors (PPIs)[1]	9 (0.1)	0 (0.0)	< 0.1

[1]This criteria considered both drugs in the association as potentially inappropriate, PPIs: proton pump inhibitors; NSAIDs: nonsteroidal anti-inflammatory drugs, TCAs: Tricyclic antidepressants, SSRIs: Selective serotonin reuptake inhibitors.

Table 8. The RECORD statement for pharmacoepidemiology (RECORD-PE) checklist of items, extended from the STROBE and RECORD statements, which should be reported in non-interventional pharmacoepidemiological studies using routinely collected health data.

Item No	STROBE items	RECORD items	RECORD-PE items	Page No
		**Title and abstract**		
1	(a) Indicate the study's design with a commonly used term in the title or the abstract.<br>(b) Provide in the abstract an informative and balanced summary of what was done and what was found.	1.1: The type of data used should be specified in the title or abstract. When possible, the name of the databases used should be included.<br>1.2: If applicable, the geographical region and timeframe within which the study took place should be reported in the title or abstract.<br>1.3: If linkage between databases was conducted for the study, this should be clearly stated in the title or abstract.	—	1
		**Introduction**		
		Background rationale		
2	Explain the scientific background and rationale for the investigation being reported.	—	—	1
		Objectives		
3	State specific objectives, including any prespecified hypotheses.	—	—	2
		**Methods**		
		Study design		
4	Present key elements of study design early in the paper.	—	4.a: Include details of the specific study design (and its features) and report the use of multiple designs if used.<br>4.b: The use of a diagram(s) is recommended to illustrate key aspects of the study design(s), including exposure, washout, lag and observation periods, and covariate definitions as relevant.	2
		Setting		
5	Describe the setting, locations, and relevant dates, including periods of recruitment, exposure, follow-up, and data collection.	—	—	2
		Participants		
6	(a) Cohort study—give the eligibility criteria, and the sources and methods of selection of participants. Describe methods of follow-up. Case-control study—give the eligibility criteria, and the sources and methods of case ascertainment and control selection. Give the rationale for the choice of cases and controls. Cross sectional study—give the eligibility criteria, and the sources and methods of selection of participants.<br>(b) Cohort study—for matched studies, give matching criteria and number of exposed and unexposed. Case-control study—for matched studies, give matching criteria and the number of controls per case.	6.1: The methods of study population selection (such as codes or algorithms used to identify participants) should be listed in detail. If this is not possible, an explanation should be provided.<br>6.2: Any validation studies of the codes or algorithms used to select the population should be referenced. If validation was conducted for this study and not published elsewhere, detailed methods and results should be provided.<br>6.3: If the study involved linkage of databases, consider use of a flow diagram or other graphical display to demonstrate the data linkage process, including the number of individuals with linked data at each stage.	6.1.a: Describe the study entry criteria and the order in which these criteria were applied to identify the study population. Specify whether only users with a specific indication were included and whether patients were allowed to enter the study population once or if multiple entries were permitted. See explanatory document for guidance related to matched designs.	Not applicabl e
		Variables		
7	Clearly define all outcomes, exposures, predictors, potential confounders, and effect modifiers. Give diagnostic criteria, if applicable.	7.1: A complete list of codes and algorithms used to classify exposures, outcomes, confounders, and effect modifiers should be provided. If these cannot be reported, an explanation should be provided.	7.1.a: Describe how the drug exposure definition was developed.<br>7.1.b: Specify the data sources from which drug exposure information for individuals was obtained.	Pages 2 - 3

			7.1.c: Describe the time window(s) during which an individual is considered exposed to the drug(s). The rationale for selecting a particular time window should be provided. The extent of potential left truncation or left censoring should be specified.<br>7.1.d: Justify how events are attributed to current, prior, ever, or cumulative drug exposure.<br>7.1.e: When examining drug dose and risk attribution, describe how current, historical or time on therapy are considered.<br>7.1.f: Use of any comparator groups should be outlined and justified.<br>7.1.g: Outline the approach used to handle individuals with more than one relevant drug exposure during the study period.	
		Data sources/measurement		
8	For each variable of interest, give sources of data and details of methods of assessment (measurement). Describe comparability of assessment methods if there is more than one group.	—	8.a: Describe the healthcare system and mechanisms for generating the drug exposure records. Specify the care setting in which the drug(s) of interest was prescribed.	Pages 2 -3
		Bias		
9	Describe any efforts to address potential sources of bias.	—	—	Not applicabl e
		Study size		
10	Explain how the study size was arrived at.	—	—	Page 3
		Quantitative variables		
11	Explain how quantitative variables were handled in the analyses. If applicable, describe which groupings were chosen, and why.	—	—	Page 3
		Statistical methods		
12	(a) Describe all statistical methods, including those used to control for confounding.<br>(b) Describe any methods used to examine subgroups and interactions.<br>(c) Explain how missing data were addressed.<br>(d) Cohort study—if applicable, explain how loss to follow-up was addressed. Case-control study—if applicable, explain how matching of cases and controls was addressed. Cross sectional study—if applicable, describe analytical methods taking account of sampling strategy.<br>(e) Describe any sensitivity analyses.	—	12.1.a: Describe the methods used to evaluate whether the assumptions have been met.<br>12.1.b: Describe and justify the use of multiple designs, design features, or analytical approaches.	Not applicabl e
		Data access and cleaning methods		
12	—	12.1: Authors should describe the extent to which the investigators had access to the database population used to create the study population.<br>12.2: Authors should provide information on the data cleaning methods used in the study.	—	Table S1 and S2
		Linkage		
12	—	12.3: State whether the study included person level, institutional level, or other data linkage across two or more databases. The methods of linkage and methods of linkage quality evaluation should be provided.	—	Page 2
		**Results**		
		Participants		

13	(a) Report the numbers of individuals at each stage of the study (eg, numbers potentially eligible, examined for eligibility, confirmed eligible, included in the study, completing follow-up, and analysed).<br>(b) Give reasons for non-participation at each stage.<br>(c) Consider use of a flow diagram.	13.1: Describe in detail the selection of the individuals included in the study (that is, study population selection) including filtering based on data quality, data availability, and linkage. The selection of included individuals can be described in the text or by means of the study flow diagram.	—	Page 3
		Descriptive data		
14	(a) Give characteristics of study participants (eg, demographic, clinical, social) and information on exposures and potential confounders.<br>(b) Indicate the number of participants with missing data for each variable of interest.<br>(c) Cohort study—summarise follow-up time (eg, average and total amount).	—	—	Pages 3 - 4
		Outcome data		
15	Cohort study—report numbers of outcome events or summary measures over time. Case-control study—report numbers in each exposure category, or summary measures of exposure. Cross sectional study—report numbers of outcome events or summary measures.	—	—	Pages 4 - 6
		Main results		
16	(a) Give unadjusted estimates and, if applicable, confounder adjusted estimates and their precision (eg, 95% confidence intervals). Make clear which confounders were adjusted for and why they were included.<br>(b) Report category boundaries when continuous variables are categorised.<br>(c) If relevant, consider translating estimates of relative risk into absolute risk for a meaningful time period.	—	—	Not applicable
		Other analyses		
17	Report other analyses done—eg, analyses of subgroups and interactions, and sensitivity analyses.	—	—	Not applcable
		**Discussion**		
		Key results		
18	Summarise key results with reference to study objectives.	—	—	Page 6
		Limitations		
19	Discuss limitations of the study, taking into account sources of potential bias or imprecision. Discuss both direction and magnitude of any potential bias.	19.1: Discuss the implications of using data that were not created or collected to answer the specific research question(s). Include discussion of misclassification bias, unmeasured confounding, missing data, and changing eligibility over time, as they pertain to the study being reported.	19.1.a: Describe the degree to which the chosen database(s) adequately captures the drug exposure(s) of interest.	Page 7
		Interpretation		
20	Give a cautious overall interpretation of results considering objectives, limitations, multiplicity of analyses, results from similar studies, and other relevant evidence.	—	20.a: Discuss the potential for confounding by indication, contraindication or disease severity or selection bias (healthy adherer/sick stopper) as alternative explanations for the study findings when relevant. **[A: Original text indicated this item was RECORD (ie, not RECORD-PE)?]**	Pages 6 - 8
		Generalisability		
21	Discuss the generalisability (external validity) of the study results.	—	—	Page 6 - 8

**Other information**				
Funding				
22	Give the source of funding and the role of the funders for the present study and, if applicable, for the original study on which the present article is based.	—	—	Page 8
Accessibility of protocol, raw data, and programming code				
22	—	22.1: Authors should provide information on how to access any supplemental information such as the study protocol, raw data, or programming code.	—	Not applicabl e

RECORD=reporting of studies conducted using observational routinely collected data; RECORD-PE=RECORD for pharmacoepidemiological research; STROBE=strengthening the reporting of observational studies in epidemiology.

*REFERENCE: **Langan SM, Schmidt S, Wing K, Ehrenstein V, Nicholls S, Filion K, Klungel O, Petersen I, Sorensen H, Guttmann A, Harron K, Hemkens L, Moher D, Schneeweiss S, Smeeth L, Sturkenboom M, von Elm E, Wang S, Benchimol EI. The REporting of studies Conducted using Observational Routinely-collected health Data (RECORD) Statement for Pharmacoepidemiology (RECORD-PE). *BMJ* 2018; 363: k3532.**
